# A specific gene expression program underlies antigen archiving by lymphatic endothelial cells in mammalian lymph nodes

**DOI:** 10.21203/rs.3.rs-5493746/v1

**Published:** 2024-12-10

**Authors:** Beth Tamburini, Ryan Sheridan, Thu Doan, Cormac Lucas, Tadg Forward, Ira Fleming, Aspen Uecker-Martin, Thomas Morrison, Jay Hesselberth

**Affiliations:** University of Colorado Anschutz Medical Campus; University of Colorado Anschutz Medical Campus; University of Colorado Anschutz Medical Campus; University of Colorado Anschutz Medical Campus; University of Colorado Anschutz Medical Campus; CU Denver Anschutz Medical Campus; University of Colorado Anschutz Medical Campus; University of Colorado; University of Colorado School of Medicine

**Keywords:** lymph node, gene expression program, antigen archiving, chikungunya virus, immunization, lymphatic endothelial cell, dendritic cell

## Abstract

Lymph node (LN) lymphatic endothelial cells (LEC) actively acquire and archive foreign antigens. Here, we address questions of how LECs achieve durable antigen archiving and whether LECs with high levels of antigen express unique transcriptional programs. We used single cell sequencing in dissociated LN tissue and spatial transcriptomics to quantify antigen levels in LEC subsets and dendritic cell populations at multiple time points after immunization and determined that ceiling and floor LECs archive antigen for the longest duration. We identify, using spatial transcriptomics, antigen positive LEC-dendritic cell interactions. Using a prime-boost strategy we find increased antigen levels within LECs after a second immunization demonstrating that LEC antigen acquisition and archiving capacity can be improved over multiple exposures. Using machine learning we defined a unique transcriptional program within archiving LECs that predicted LEC archiving capacity in mouse and human independent data sets. We validated this modeling, showing we could predict lower levels of LEC antigen archiving in chikungunya virus-infected mice and demonstrated *in vivo* the accuracy of our prediction. Collectively, our findings establish unique properties of LECs and a defining transcriptional program for antigen archiving that can predict antigen archiving capacity in different disease states and organisms.

## Introduction

Viral antigens persist within lymph nodes following viral infection^1–5^. Persisting antigen, in the case of influenza virus infection, can recruit memory T cells, promoting protection against future infections with the same virus^1–3,5–7^, demonstrating that persisting viral antigens may be an important mediator of immune memory responses. We demonstrated that protein subunit immunization, administered with a Toll-like receptor (TLR) agonist subcutaneously, also initiates persistence of the protein antigen in the draining lymph node (dLN)^8–11^. Similar to virus-derived persisting antigen, the vaccine-derived persisting antigen improved T cell memory^8,10^. We termed this process of durable antigen retention “antigen archiving”^10^.

Lymphatic endothelial cells (LEC)s interact with antigens and virus as they traffic through lymphatic capillaries to lymphatic collectors before entering the lymph node sinus and the fibroblastic reticular cell (FRC) conduit system. These two cell types, LEC and FRC, along with blood endothelial cells (BEC), comprise a larger non-hematopoietic subset of cells within the LN called lymph node stromal cells (LNSC). In recent years many new LNSC subtypes have been defined using single-cell RNA-seq^11–14^, propelling our understanding of the diversity of LNSCs and their contributions to LN function. Despite these advances, the functional roles of LNSC subpopulations are poorly defined. To begin to understand how LNSCs archive antigens, we previously quantified antigen levels across different LEC subsets using a protein antigen conjugated to a stabilized DNA tag and tracked antigen distribution after immunization using single cell sequencing and spatial transcriptomics^11^.

While LECs can present peripheral tissue antigens to promote peripheral tolerance, archived antigens are exchanged to migratory conventional dendritic cells (cDC1 and 2) to promote immunity. Consistent with live imaging and antigen presentation assays, antigen-DNA conjugates were only found in CCR7hi migratory cDCs^11^. Thus, as migratory DCs traffic from the skin to the dLN, they interact with antigen bearing LECs, acquiring antigens to improve the effector function of memory CD8 T cells^9–11^. Indeed, some antigen is acquired from LECs that undergo apoptosis during LN contraction^8,9^. To understand whether LEC apoptosis or LEC-DC antigen exchange could be manipulated, we studied the consequences of immune insult to antigen held within LECs and found that a subsequent inflammatory stimulus within the archiving timeframe yielded increased T cell effector responses compared to those that did not have a secondary stimulus. These improved responses depended on T cell receptor (TCR) stimulation, but were independent of bystander activation^8^. Protection from these boosted memory CD8 + T cells was durable and robust, establishing a model wherein cell-mediated immunity can be manipulated by the presentation of previously archived antigens. Collectively, these studies demonstrate a novel role for the LNSC and particularly LECs, in promoting protective T cell immunity at late time points after immunization.

LECs acquire multiple types of protein antigens^8–10^ including purified chikungunya (CHIKV) viral E2 glycoprotein, albumin, ovalbumin, SARS-Co-V-2 receptor binding domain (RBD)^8^, influenza nucleoprotein (NP), herpes simplex virus glycoprotein B (HSVgB), and vaccinia viral proteins post-infection^10^. In addition to acquisition and retention of foreign protein antigens, LECs may support viral replication, such as Kaposi’s sarcoma associated herpes virus^15^. Recently, single cell mRNA sequencing of LNSCs during CHIKV infection indicated that subsets of LECs that express the scavenger receptor macrophage receptor with collagenous structure (MARCO) may support CHIKV RNA replication^16^, consistent with another study showing MARCO-dependent internalization of CHIKV by LN LECs^17^. CHIKV RNA is also detectable in FRCs, which express the CHIKV entry receptor Mxra8^18,19^. Although CHIKV infection causes LN disorganization and impaired CD8^+^ T cell responses^17,20^, exploiting this mechanism of viral targeting could lead to a better understanding of the functions of distinct LNSCs. Confirmation of these entry mechanisms used single cell RNA sequencing to detect very low levels of CHIKV RNA within MARCO-expressing LECs^16,17^.

In our previous studies of antigen-DNA conjugates, antigen levels were assessed 2 days and 2 weeks post-immunization^11^. More recently^8^, we found that the protective capacity of archived antigens was maintained at longer time points (> 2 weeks post-immunization), especially when there was a secondary inflammatory stimulus that caused the release of archived antigens to stimulate memory T cells. However, there are still unanswered questions about how LNSCs archive antigens, including the duration and maintenance of archived antigens within LECs, whether LECs acquire and retain multiple antigens following sequential immunizations, and whether LNSC are intrinsically programmed to archive antigens, or whether they learn to archive additional antigens after acquisition.

## Results

### Antigen persists in distinct cell populations within the lymph node

To study the dissemination of antigens in the draining lymph node after vaccination, we previously developed a scRNA-seq approach to quantify antigen levels *in vivo*^10^ involving conjugation of a model antigen, ovalbumin (ova), to a DNA barcode containing phosphorothioate linkages (psDNA). Using this tool, we showed that immunization with ova-psDNA and vaccinia virus (VV) induces robust antigen archiving^9,11^. We identified populations of LNSCs that retain antigen for 14 days post-vaccination and gene expression patterns associated with antigen archiving^11^. The ability of a LEC to archive antigen could be influenced by two separate processes: 1) antigen uptake, which likely occurs through endocytic pathways, and 2) antigen retention, which would be influenced by the cellular compartment where the archived antigen is stored. However, our previous studies^11^, lacked the temporal resolution to differentiate between these two processes. Here we sought to evaluate the dynamics of antigen uptake and archiving by performing an extended 42 day post-immunization time-course.

**Figure 1:**
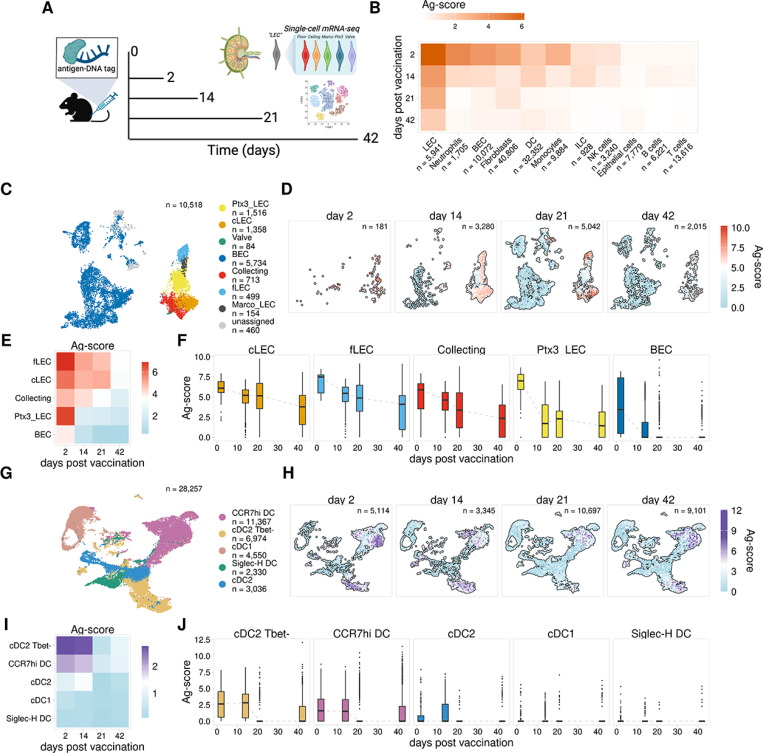
Antigen persists in discrete cell populations within the lymph node A. Experimental design B. Mean Ag-score is shown for each cell type for 2, 14, 21, and 42 days post vaccination. C. UMAP projection shows LEC subsets. D. UMAP projections show Ag-scores for LEC subsets for each timepoint. E. Mean Ag-score is shown for LEC subsets for each timepoint. F. Ag-scores are shown for each timepoint for each LEC subset. G. UMAP projection shows DC subsets. H. UMAP projections show Ag-scores for DC subsets for each timepoint. I. Mean Ag-score is shown for DC subsets for each timepoint. J. Ag-scores are shown for each timepoint for each DC subset.

We immunized mice subcutaneously with ova-psDNA + VV to both track exogenous antigen and induce robust LN expansion and antigen archiving^8,11^ and performed scRNA-seq on CD45- cell populations from the dLN at 21 and 42 days post-vaccination. In a separate cohort of mice immunized at the same time, we sorted dLN into four populations, which were enriched for CD11c+, CD11c-CD11b+, B220 + and all other cells. We performed scRNA-seq using these sorted populations mixed a ratio of 4:4:1:1, respectively^11^. We then compared antigen levels across a time-course using our published scRNA-seq data collected 2 and 14 days post-immunization^11^ (Fig. 1A). To accurately compare cell populations across the four time points, we integrated the scRNA-seq data for each time point and re-annotated the cell types present in each sample using a previously published automated approach^21^ (Figure S1A). To compare levels of antigen across our datasets we calculated background-corrected antigen scores (Ag-score) for each cell, normalizing counts by the total antigen counts for the sample, subtracting the estimated background signal based on counts present in B and T cells, and log-transforming (see methods). In support of our previous work, we detected the highest antigen levels within lymphatic endothelial cells (LECs), followed by lower levels in neutrophils, blood endothelial cells (BECs), fibroblasts, monocytes, and dendritic cells (DCs) (Fig. 1B, S2A–B). Levels of antigen were highest 2 days post-vaccination (Fig. 1B). In all cell types identified, except LECs, antigen levels dropped sharply by day 14 and were largely undetectable by day 42. LECs maintained high levels of antigen throughout the 21 day time-point, however by day 42 antigen levels were substantially reduced, though still higher than all other cell populations. These results support our previous work showing that LECs are the predominant cell population that acquires and archives antigens in the draining lymph node^11^.

To investigate the dynamics of antigen acquisition and archiving across different LEC subsets, we next annotated different endothelial cell populations using previously published data^11,14^ (Figure S1B, C). Using this approach, we identified subsets of collecting and valve LECs that form the afferent and efferent collecting vessels of the lymph node (collecting LECs) (Fig. 1C, S1B–C), as well as floor (fLEC) and ceiling (cLEC) LECs of the subcapsular sinus (SCS), which express the adhesion molecule Madcam1 and the atypical chemokine receptor Ackr4, respectively (Fig. 1C, S1B,D). SCS LECs are the first LEC populations that encounter lymph-borne antigens after entry into the lymph node and we previously showed that these populations have the highest levels of antigen 14 days post-immunization^11^. We also identified LEC subsets from the medullary and paracortical sinuses, which are distinguished by the expression of the scavenger receptors MARCO (Marco LECs) and Ptx3 (Ptx3 LECs)^11,14,16,17^ (Fig. 1C, S1B,D). Analysis of antigen levels over time for each LEC population revealed that at 2 days post-immunization, LECs show universally high levels of tagged antigen (Fig. 1D–F, S2C). These findings indicate that immediately following a protein subunit immunization, antigen is dispersed throughout the LN, including in Marco and Ptx3 LECs, which are among the last populations to encounter antigen, based on the location spatial locations of these subsets as identified by others^14^. However, antigen levels at later times after immunization showed significant differences in antigen archiving ability. We detected high antigen levels in valve LECs, cLECs, fLECs, collecting LECs, and Marco LECs through 21 days post-immunization, while antigen levels drop significantly by day 14 in Ptx3 LECs (Fig. 1D–F, S2C). These results indicate that differences in antigen archiving are not due to LEC localization within the lymph node or efficiency of antigen uptake, as we observe high amounts of antigen within all LEC populations 2 days after immunization, and instead suggest that the dynamics of antigen archiving are a key determinant of overall antigen levels.

The protective capacity conferred by antigen archiving is dependent on antigen exchange with migratory DCs, which present archived antigen peptides to memory CD8 T cells^8,9^. To explore the dynamics of antigen exchange between LECs and different DC subsets, we integrated the DC datasets for each time point and annotated specific DC populations using previously published data^11,22,23^, identifying conventional (c) DC1 and DC2 populations along with CCR7hi migratory cDCs and Siglec-H-expressing plasmacytoid DCs (Fig. 1G, S1C.E). Comparison of antigen levels between these populations showed cDC2 Tbet- and CCR7hi DCs had the highest antigen levels 2 days post-vaccination, with other subsets having minimal levels at any time point (Fig. 1H–J, S2D). The CCR7hi and Tbet- DC subsets both showed consistent levels of antigen through day 14, with levels dropping sharply by day 21. These findings are consistent with other reports demonstrating the length of time DCs remain viable after activation^24^. Therefore, we suggest that any antigen remaining within the DC populations beyond 2–3 weeks is likely a result of DC acquisition of antigen from other cell types. Consistent with our published data that migratory DCs are required for antigen exchange, it is likely the antigen positive DCs remaining have acquired antigen from the only cell type with antigen at these late time-points, LECs.

### Identification of gene signatures associated with antigen archiving by LECs

**Figure 2:**
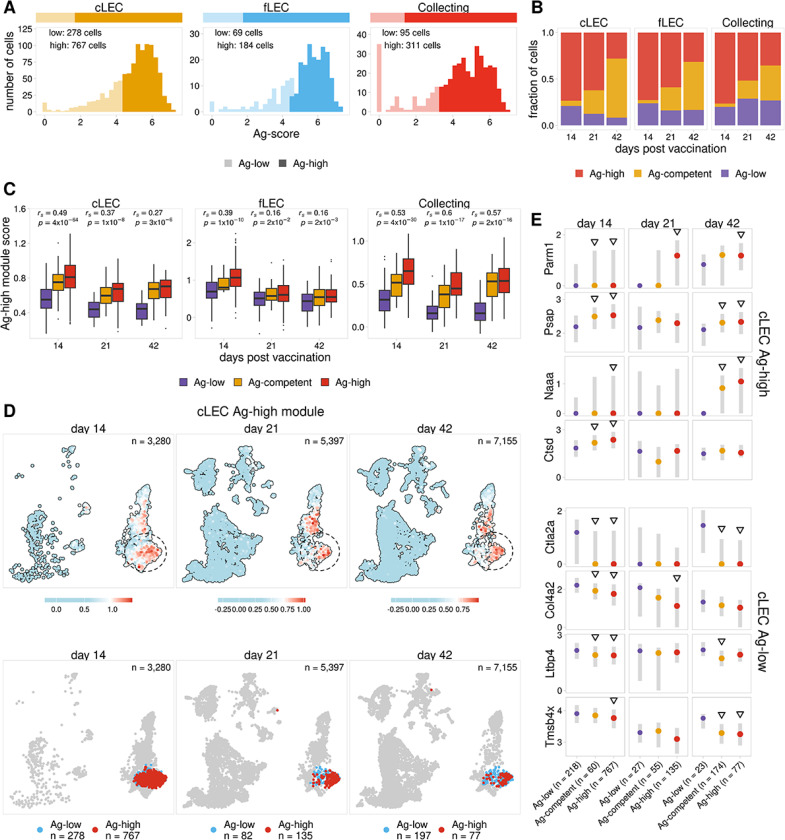
Identification of an antigen archiving gene signature A. Ag-score is shown for day 14 Ag-high and -low cells identified for LEC subsets with the highest Ag-score. B. The fraction of cells predicted to be Ag-competent is shown for each LEC subset 14, 21, and 42 days post vaccination. C. Ag-high module score is shown for Ag-low, Ag-high, and predicted Ag-competent LECs. The Spearman correlation between Ag classes and Ag-high module score is shown for each timepoint. One-sided p values were calculated and adjusted using Benjamini-Hochberg correc-tion. D. UMAP projections show cLEC Ag-high module scores for each timepoint. E. The expression of select genes from the Ag-high (top four) and Ag-low (bottom four) gene mod-ules is shown for cLECs. Genes identified as top predictors for multiple LEC subsets are shown. Triangles indicate the gene is differentially expressed when compared to Ag-low cells. P values were calculated using a one-sided Wilcoxon rank sum test with Benjamini-Hochberg correction.

Comparison of antigen levels throughout the time course revealed significant differences in antigen archiving between LEC populations. We next identified gene expression signatures that might influence the ability of different LEC subsets to archive antigen. We previously identified genes associated with caveolin-mediated endocytosis that are expressed more strongly in LECs with higher levels of archived antigen at day 14^11^. We sought to leverage our new scRNA-seq data to expand on this idea and further characterize gene expression programs underlying antigen archiving. We first identified LECs with high levels of antigen (Ag-high) at the day 14 time point by performing k-means clustering separately for each LEC subset using the calculated antigen scores (Fig. 2A). To ensure consistent classification of Ag-high cells across the time points, we used the day 14 antigen score cutoffs to classify antigen-high (Ag-high) cells in the day 21 and 42 time points.

To investigate gene expression patterns underlying antigen retention, we next identified genes that were differentially expressed between Ag-high and -low populations. Using these genes, we trained separate random forest classifiers using day 14 cLECs, fLECs, and collecting LECs (Table S1), which showed the highest levels of archived antigen at this time point. Although valve and Marco LECs also exhibited higher levels of antigen throughout the time course, the relatively small number of cells identified in these subsets precluded them from downstream analysis (Figure S2C). Using these models, we identified genes most predictive of whether an LEC from one of these subsets would archive high or low amounts of antigen. We then grouped these genes into distinct Ag-high and Ag-low gene programs based on whether the gene was up- or down-regulated in Ag-high cells, respectively (Table S2). We find that the gene programs identified for each LEC subset are distinct and have relatively few overlapping genes (Figure S3A). To characterize cellular processes potentially influencing antigen archiving, we identified gene ontology terms for the Ag-high and -low gene programs for each LEC subset (Figure S3B–C, Table S3). From this analysis, we find that the Ag-high programs are enriched for genes suggestive of increased endosomal-lysosomal function (Figure S3B, Table S3), and this is most significant for cLECs and fLECs. This includes lysosome factors with broad roles in protein homeostasis (Ctsd^25^, Grn^26^, Lgmn^27^, Prcp^28^), lipid metabolism (Psap^29^), and lysosome acidification (Atp6v1h^30^ Atp6ap1^31^). In addition, expression of the clathrin adaptor protein Dab2^32^ may indicate increased endocytic activity. The collecting LEC Ag-high module also includes genes related to endosomal-lysosomal function (Psap^29^, Atp6v1h^30^), however, this module is more strongly associated with broader endothelial cell activation. This includes genes related to cell migration (F11r^33^, Rock2^34^), inflammatory signaling (Map3k1^35^, Nfat5^36^), cell proliferation (Tgfa^37^), and extracellular matrix (ECM) remodeling (Tgm2^38^). Analysis of the Ag-low gene programs show enrichment of genes associated with established structural and functional roles of LECs (Figure S3C, Table S3). This includes genes that promote lymphangiogenesis (Relb^11^, Hmgb1^39^), ECM factors that provide structural support for lymphatic vessels (Col4a2, Lamc1, Lama4, Nid2, Fn1)^40,41^, and genes involved in immune cell trafficking (Ccl21a^42^).

To further investigate how these gene programs could impact antigen archiving, we next assessed whether Ag-high genes were induced in response to an immune stimulus. We compared each time point after immunization with previously published scRNA-seq data from sorted LNSCs of naïve mice^17^. We found that these programs are robustly expressed in LECs from naïve mice and expression persists 42 days post-vaccination when most archived antigen has been released or degraded (Figure S3C–D). These finding suggest that the identified gene programs are expressed by most LECs regardless of inflammatory state, and are not specifically induced in response to immune activation. Together these results support a distinct program where Ag-high LECs, which have a transcriptome shifted towards increased endo-lysosomal capacity, are equipped to acquire and process a higher volume of internalized material (ie foreign protein antigens). The high levels of antigen acquired by LECs are therefore likely to contribute to the length of antigen duration, consistent with prior work^10^.

Continued expression of the Ag-high gene program after 21 and 42 days post immunization (Figure S3D) suggests there are still LECs that are competent to archive antigen even though most antigen has been degraded or released by these time points. To further investigate these potential archiving-competent cells, we attempted to identify LEC populations that would be predicted to archive antigen at the 21 and 42 day time points, based on the classification models trained using the day 14 time point. From this analysis we classified cells into three groups: 1) Ag-low has low amounts of archived antigen based on quantification of antigen scores, 2) Ag-competent has low amounts of antigen but is predicted to have high antigen levels by our classification models, and 3) Ag-high has high levels of antigen. We find that at day 21 and 42 most cLECs, fLECs, and collecting LECs that have low levels of antigen are predicted to be Ag-competent (Fig. 2B, S3F). To assess the accuracy of these predictions, we compared expression of the Ag-high and Ag-low gene modules derived from our day 14 classification models. As expected, we found that the predicted Ag-competent cells have higher expression of the Ag-high module and reduced expression of the Ag-low module when compared with Ag-low cells (Fig. 2C, S3G). Furthermore, by comparing Ag-low, Ag-competent, and Ag-high cells, we found a notable correlation between these classes and the expression of the Ag-high and -low gene programs (Fig. 2C, S3G). We saw that LECs that retain high amounts of antigen at the 21 and 42 day time points generally show the strongest and weakest expression of the Ag-high and -low programs, respectively (Fig. 2C–E, S3G–I). To summarize, we were able to identify gene signatures (Figure S3A–B, Table S2) expressed in LECs from naive mice (Figure S3D–E) that are predictive of the capacity of a cell to archive antigen (Fig. 2B, S3F, Table S1) and correlate with the duration of antigen retention (Fig. 2C, S3G).

### Antigen archiving by LECs is enhanced by sequential immunization

**Figure 3:**
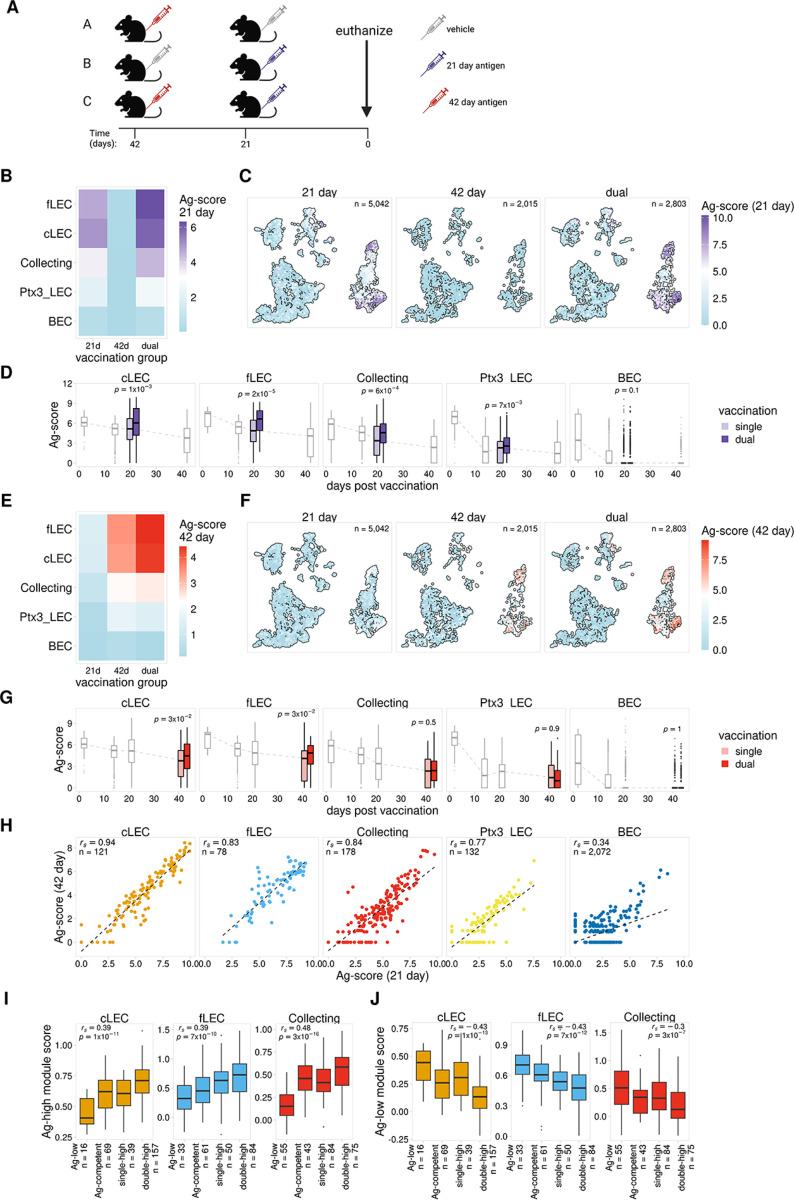
Antigen archiving is enhanced by sequential vaccinations A. Experimental design B. Mean 21 day Ag-score is shown for LECs from mice that received a single vaccination (21 day or 42 day) or dual vaccination (21 day and 42 day). C. UMAP projections show 21 day Ag-scores for LEC subsets for single and dual vaccinations. D. Prior vaccination enhances antigen archiving. Ag-score is shown for single and dual vaccina-tions for the 21 day timepoint for each LEC subset. Other timepoints are shown in grey. P val-ues were calculated using a one-sided Wilcoxon rank sum test with Benjamini-Hochberg cor-rection. E. Mean 42 day Ag-score is shown as described in A. F. UMAP projections show 42 day Ag-scores for LEC subsets as described in B. G. Successive vaccinations enhances retention of previously archived antigen. Ag-score is shown for the 42 day timepoint as described in C. H. 21 day and 42 day Ag-score is compared for LEC subsets. I. Ag-high module scores described in Figure 2 are shown for LECs that archived antigens from both vaccinations (double-high), from only one vaccination (single-high), or have low levels of both antigens (Ag-low). Module scores are shown for the corresponding LEC subset. P values were calculated using a one-sided Wilcoxon rank sum test with Benjamini-Hochberg correc-tion. J. Ag-low module scores are shown for LEC subset as described in H.

We have previously shown that a subsequent immune challenge results in the presentation of previously archived antigens, which in turn boosts protective immunity^14^. To expand on these results, we next investigated how the dynamics of antigen archiving are impacted by sequential immunizations administered within the archiving time frame. We compared mice from three vaccination groups, A) received one vaccination 42 days prior to harvest, B) received one vaccination 21 days prior to harvest, and C) received both vaccinations, 42 and 21 days prior to harvest (Fig. 3A). For each group we immunized WT mice with ova-psDNA + VV subcutaneously, harvested dLN, and performed scRNA-seq on LN populations as described above. To separately quantify archived antigen originating from the 21 day (purple syringe) and 42 day (red syringe) time points, the psDNA tags for each immunization contained unique barcodes (Fig. 3A).

To first investigate how previously archived antigen affects archiving during a future immune challenge, we compared levels of the 21 day antigen for LECs from group B, which only received the second immunization (21 day), and group C, which received both immunizations. Consistent with our previous results (Fig. 1), the LEC subsets with the highest levels of antigen in the dual vaccinated mice (group C) are cLECs, fLECs, and collecting LECs (Fig. 3B). As expected, we also observed minimal background levels of the 21 day antigen for mice that did not receive the 21 day immunization (Fig. 3B–C). Comparison of the 21 day antigen levels for single vs dual vaccinated mice revealed elevated levels of archived antigen in mice that received both the 42 and 21 day immunizations (Fig. 3B–D, S4A). This suggests that LECs that previously archived antigen have enhanced uptake and/or retention of antigens from a future immunization. Whether this function of increased antigen uptake is permanent and the result of an altered transcriptional program caused by antigen acquisition or inflammation is still unclear.

Using this dual vaccination model, we next tested the inverse scenario and asked how successive immunizations affect retention of previously archived antigen. To do this we compared levels of the 42 day antigen for mice that only received the first vaccination (group A) vs mice that received both vaccinations (group C) (Fig. 3A). Similar to the results for the 21 day antigen, we saw significantly higher levels of the 42 day antigen in dual vaccinated mice, suggesting prolonged retention of the previously archived antigen (Fig. 3E–G, S4B). Taken together, these results indicate that sequential vaccinations promote an overall increase in antigen archiving by LECs.

To further investigate the dynamics of antigen uptake and retention, we next asked whether increased antigen levels observed in dual vaccinated mice was due to distinct groups of LECs acquiring each antigen. Evaluation of LECs from the 42 day time point indicated a substantial fraction of cells with low levels of antigen that expressed a gene program predictive of increased antigen archiving ability (Ag-competent, Fig. 2B). This represents a cell population that could be well suited to take up new antigen during a future immune challenge. From this observation, we predicted that in dual vaccinated mice we would observe two distinct groups of Ag-high cells, one primarily containing antigen from the first vaccination (42 day) and a separate group containing antigen from the second vaccination (21 day). To test this model, we compared levels of each tagged antigen in the dual vaccinated mice. Contrary to our prediction, we did not observe distinct groups of LECs that exclusively archived the different antigens, but instead found a strong correlation between levels of the tagged antigens for each LEC subset, with cells containing high levels of the first antigen (42 day) also showing high levels of the second antigen (21 day) (Fig. 3H, S4D). We confirmed these findings *in vivo* using flow cytometry of fluorescently labeled ovalbumin with polyI:C/aCD40, and similarly found that the same LECs from draining LNs of mice immunized 1 week apart were positive for both antigens delivered sequentially (Figure S5), indicating there are populations of cells within each LEC subset that are particularly well suited to archive antigen and will acquire antigens from repeated immune challenges.

Finally, we next asked whether there were distinct gene expression signatures associated with cells that archived multiple antigens in the dual immunized mice. We analyzed expression of the previously identified Ag-high and Ag-low gene modules (Fig. 2, Table S2) to determine if these programs might also influence the ability of a LEC to archive antigens from multiple vaccinations. We identified LECs from the dual immunized mice that had high levels of each antigen using the antigen score cutoffs derived from the day 14 time point (Fig. 2A) and predicted Ag-competent cells using our previously described classification models (Fig. 2, S3, Table S1). We then identified LECs with high levels of both antigens (double-high), high levels of only one antigen (single-high), low levels of both antigens but predicted to be Ag-competent (Ag-competent), and low levels of both antigens (Ag-low). Comparison of these groups reveals a significant correlation with expression of the Ag-high and Ag-low gene programs (Fig. 3I–J, S4E–F). We found that the double-high cells, which are the most proficient at archiving, had the highest expression of the Ag-high gene program and the lowest expression of the Ag-low gene program for each LEC subset (Fig. 3I–J, S4E–F). These findings suggest that we have identified at least some of the gene programs that promote antigen archiving during an initial immunization and that these programs also function to promote archiving during sequential immune challenges.

### Antigen exchange with DCs correlates with levels of antigen archiving

We have previously shown that to enhance protective immunity, LECs exchange archived antigen with migratory DCs, which present to memory CD8 + T cells^9,10^. To further investigate antigen exchange between LECs and DCs, we assessed the spatial localization of antigen in the dLN using two spatial transcriptomics platforms, Xenium (10x Genomics) and GeoMx DSP (Nanostring). We repeated our dual vaccination experiment and immunized mice with ova-psDNA for 21 days, 42 days, or sequentially 21 days apart using ova-psDNA conjugates containing unique barcodes with VV (Fig. 3A). We excised draining popliteal LNs at each time point, fixed with formalin, and embedded into paraffin. Tissue sections were cut and analyzed with the Xenium and GeoMx DSP platforms using probes for the mouse transcriptome and custom probes recognizing the 21 day and 42 day antigen barcodes.

**Figure 4:**
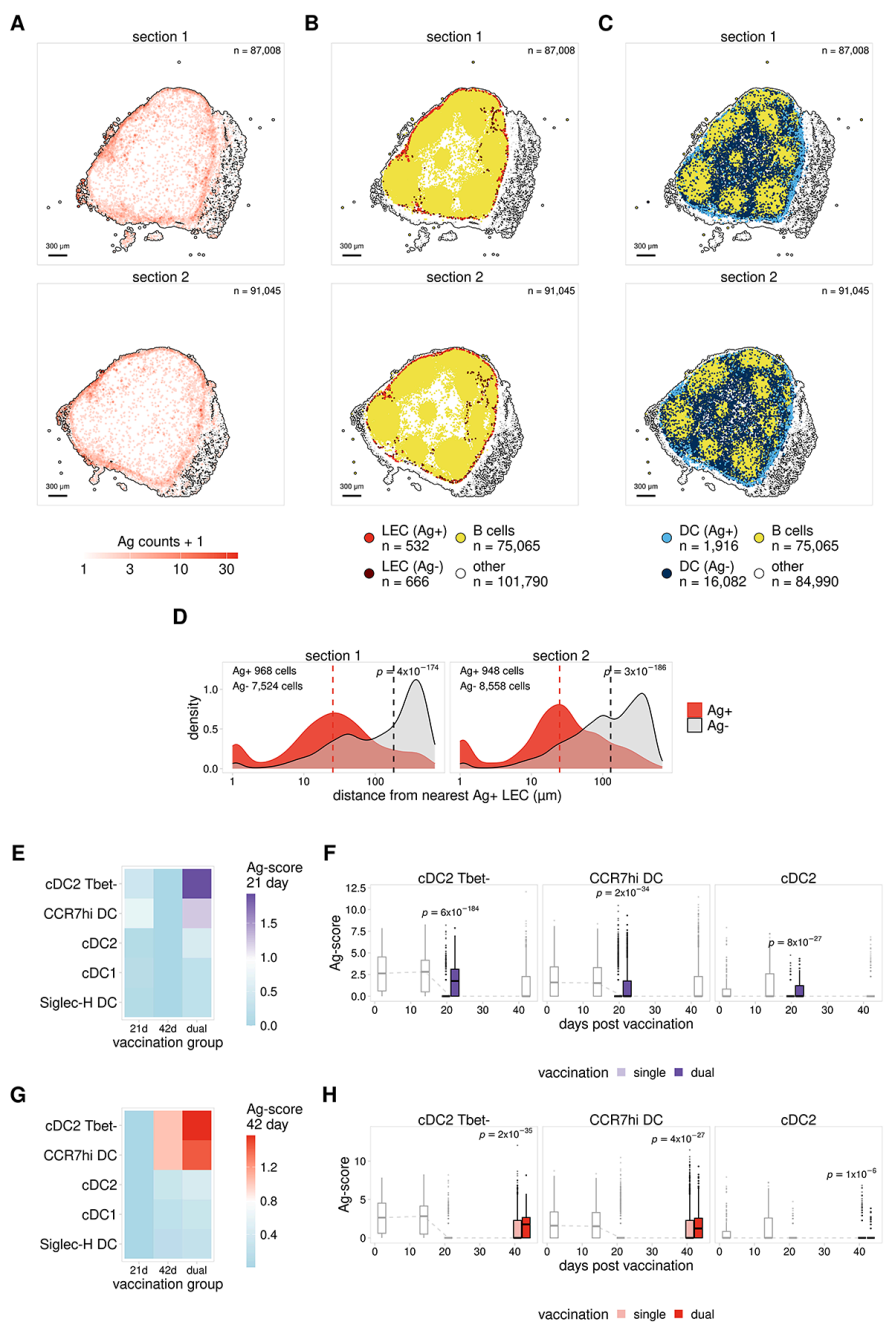
Antigen exchange with DCs correlates with levels of antigen archiving A. Antigen counts are shown for adjacent LN tissue sections from a dual-immunized mouse ana-lyzed with the Xenium platform. B. Localization of Ag+ and Ag- cells is shown for LECs for tissue sections from A. C. Localization of Ag+ and Ag- cells is shown for DCs for tissue sections from A. D. The distance to the closest Ag+ LEC is shown for Ag+ and Ag- DCs for tissue sections from A. E. Mean 21 day Ag-score is shown for DCs from mice that received a single vaccination (21 day or 42 day) or dual vaccination (21 day and 42 day). F. Ag-score is shown for single and dual vaccinations for the 21 day timepoint for DC subsets with highest Ag-score. Other timepoints are shown in grey. P values were calculated using a one-sided Wilcoxon rank sum test with Benjamini-Hochberg correction. G. Mean 42 day Ag-score is shown for DCs from mice that received single or dual vaccination as described in F. H. Ag-score is shown for single and dual vaccinations for the 42 day timepoint as described in G.

We obtained Xenium data for a total of 6 individual LNs, which included 2 lymph nodes for each immunization group (Fig. 3A). For each LN we assayed two adjacent tissue sections for a total of 4 sections per immunization group. LN sections from one of the dual immunization samples came from the outer edge of the LN and were excluded from downstream analysis. To classify cell types present in these sections we generated a reference based on the cell type annotations from our scRNA-seq data (Figure S1A) and compared this to expression patterns for the mouse genes included in our Xenium probe panel (see methods). Using this approach we identified large populations of B cells, T cells, and DCs, along with smaller populations of stromal cells including LECs, BECs, and fibroblasts (Figure S6A). Analysis of antigen probe counts indicated that samples from mice receiving a single immunization showed only background levels of antigen (Figure S6B). However, consistent with our previous findings (Fig. 3, S4) LN sections from mice that received both immunizations showed enhanced antigen signal that was specifically localized to the outer subcapsular sinus (Fig. 4A). To characterize cells harboring antigen, we next used the LN sections from our dual immunized sample and classified Ag + cells as any cell with at least 1 antigen count for the 21 day or 42 day antigen probes. From this analysis, we find that LECs show the strongest enrichment of Ag + cells (Fig. 4B, S6C). This supports our previous scRNA-seq results showing that LEC populations making up the ceiling and floor of the subcapsular sinus (cLECs and fLECs) contain the highest levels of archived antigen (Fig. 1D–F).

We next asked whether the localization of DCs to different regions of the LN influenced the amount of antigen acquisition by DCs. Assessment of the localization of Ag + and Ag- DCs within the dual immunized sample shows that Ag + DCs localize in and around the SCS, while Ag- DCs are broadly distributed throughout the LN (Fig. 4C). To further quantify this result, for each DC we calculated the distance to the closest Ag + LEC. We find that Ag + positive DCs are localized in significantly closer proximity to Ag + LECs than Ag- DCs (Fig. 4D). This observation supports our previous results^8,9^, and suggests that antigen archived by LECs in the SCS is handed off to DCs located near the SCS or passing through the SCS.

The close proximity of Ag + LECs and DCs is further supported by analysis of the single and dual immunized samples (Fig. 3A) using the GeoMx DSP platform. For these samples, we obtained GeoMx data for a total of 15 individual LNs divided across two slides (Figure S6A). Distinct regions (composed of > 500 cells on average) within each dLN were selected for analysis, including the SCS, medulla, and cortex (Figure S7B). The GeoMx platform allows each selected region to be further segmented based on fluorescent staining. To distinguish between different cell populations, each region was segmented based on antibody staining for Lyve1 (LECs), CD11c (DCs), and CD4 (T cells) (Fig. 4B).

To identify regions with the highest levels of antigen, we next compared the normalized antigen signal for probes targeting the 21 day and 42 day antigens. To compare antigen signals for each region, we pooled regions from the single and dual immunized mice. We then scaled the normalized signal separately for each antigen to allow the 21 and 42 day antigen signals to be compared together. We find that antigen levels for both Lyve-1 + and CD11c + segments are highest within the SCS regions (Figure S7C). In addition, there is a strong correlation between antigen signals for Lyve-1 + and CD11c + segments within each region, and regions with higher antigen levels in LECs (Lyve-1+) also show higher antigen levels in DCs (CD11c+) (Figure S7D). Taken together, our spatial transcriptomics results (Fig. 4A–D, S6,S7). Taken together, our spatial transcriptomics results (Fig. 4A–D, S6, S7) suggest that the amount of antigen exchanged with DCs is directly dependent on the amount of antigen archived by proximal LEC populations where SCS LECs archive the most antigen and therefore DCs located near the SCS also have high amounts of antigen.

We next asked if there was increased antigen uptake by DCs in dual vaccinated mice, which showed higher levels of archived antigen in LEC populations (Fig. 3A–G, S4B). Due to the poor antigen signals obtained for single-immunized mice analyzed with the Xenium platform (Figure S6B), and the low number of regions obtained for dual immunized mice analyzed with the GeoMx platform (Figure S7A–B), we were not able to compare DC antigen signals using these spatial transcriptomics approaches. Instead, we used our scRNA-seq data to analyze DC populations from single- and dual-immunized mice (Fig. 3A). In support of our previous results (Fig. 1H–J, S2D), we found that CCR7^hi^ and cDC2 Tbet-populations had the highest antigen levels in the dual-vaccinated samples. In addition, similar to LECs (Fig. 3B–G, S4A), we found that DCs from dual vaccinated mice had significantly higher antigen levels than mice that only received one of the immunizations (Fig. 4E–H, S4A,C). These results in conjunction with our spatial transcriptomics data (Fig. 4A–D, S6, S7), support a model wherein successive vaccinations enhances antigen uptake and archiving, which in turn results in increased exchange of antigen with proximal or migrating DC populations.

### Antigen archiving is impaired during CHIKV infection

**Figure 5:**
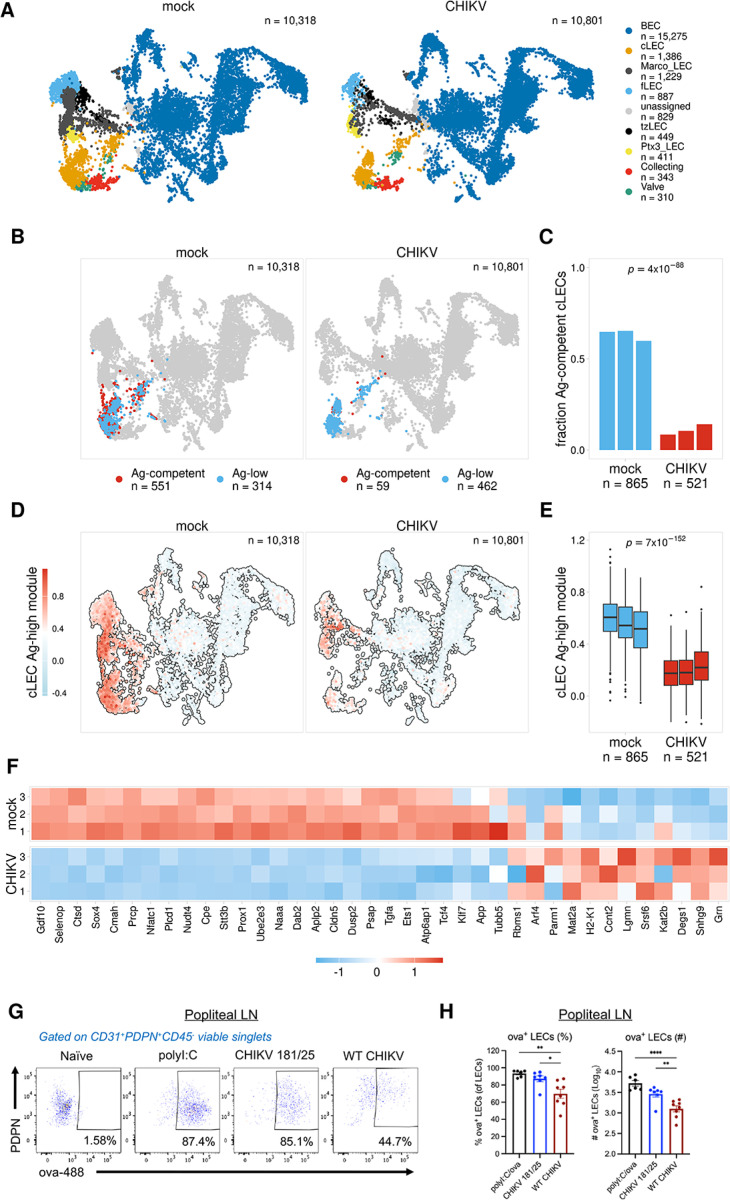
Antigen archiving is impaired during CHIKV infection A. UMAP projections show LEC subsets for mock and CHIKV-infected mice. B. UMAP projections show predicted Ag-competent cLECs for mock and CHIKV-infected mice. C. The fraction of predicted Ag-competent cLECs is shown for mock and CHIKV-infected mice for each biological replicate. P values were calculated using Fisher’s exact test with Benjamini-Hochberg correction. D. UMAP projections show cLEC Ag-high module scores for mock and CHIKV-infected mice. E. cLEC Ag-high module scores are shown for mock and CHIKV-infected mice for each biological replicate. P values were calculated using a two-sided Wilcoxon rank sum test with Benjamini-Hochberg correction. F. Expression of the cLEC Ag-high gene module is shown for mock and CHIKV-infected mice for cLECs from each biological replicate. G. Representative flow cytometry plots showing ova488+ LECs. H. Percentage and number of ova488+ LECs from mice injected with ova-488 (10μg) 24 hours post 103 plaque forming units (PFU) of CHIKV WT or strain 181/25 inoculated in the footpad and euthanized 24 hours after ova-488 injection. For polyI:C group mice were immunized with ova-488 (10μg) and polyI:C (5μg) and euthanized 24 hours later (no CHIKV infection). *p<0.05, **p<0.01, ****p<0.0001, by one-way ANOVA with Turkey’s multiple comparison test.

Using the classification models we trained the main Ag + LEC subsets, we identified cells that are predicted to be archiving-competent (Ag-competent) based on the expression of distinct gene programs (Fig. 2, Table S2). To further test the utility of these models, we asked if we could predict differences in the antigen archiving capability of LECs from naive mice and mice infected with CHIKV, which we previously reported impairs LN antigen acquisition^16^. We used previously published scRNA-seq data of murine LNSCs 24 hours after infection with the arthritogenic alphavirus CHIKV^16,17^. CHIKV infection in mice disrupts the structure and function of the draining lymph node^17,20^ and induces a proinflammatory and antiviral response in LECs^16^. Importantly, by 72 hours post-infection with WT CHIKV, antigen acquisition by LN LECs is impaired^16^. Using scRNA-seq data for LNSCs at 24 hours post CHIKV infection (Fig. 5A), we asked if we could use our antigen archiving classification models to demonstrate whether CHIKV infection would also impede antigen archiving at 24 hours post infection.

The scRNA-seq data for CHIKV-infected mice includes similar subsets of LECs, including cLECs, fLECs, and collecting LECs (Fig. 5A). To evaluate antigen archiving during CHIKV infection, we predicted archiving ability for cLECs, fLECs, and collecting LECs from mock- and CHIKV-infected samples using the classification models previously trained for each of these subsets (Fig. 5B–C, S8C–D). To first assess the accuracy of these predictions, we compared expression of the Ag-high and Ag-low gene modules derived from each model (Fig. 2, Table S2). As expected, there was overall higher expression of the Ag-high gene module and lower expression of the Ag-low gene module for LECs predicted to be archiving-competent (Ag-competent) (Figure S7A). We next compared the fraction of Ag-competent cells for the mock- and CHIKV-infected samples. We found that for the combined population of antigen archiving LECs (cLECs, fLECs, collecting LECs), there was a significant reduction in the overall fraction of Ag-competent cells for CHIKV-infected mice (Figure S8C, with cLECs showing the largest reduction (Fig. 5B–C, S8C–D). Comparison of expression differences for the Ag-high and Ag-low gene modules for mock- and CHIKV-infected mice further supported these findings. For cLECs we found a significant reduction in expression of the Ag-high module and an increase in expression of the Ag-low module during CHIKV infection (Fig. 5D–F, S8B). However, fLECs and collecting LECs both had less pronounced changes in the fraction of Ag-competent cells and in the expression of these modules. fLECs showed some increase in Ag-competent cells and a corresponding increase and decrease in the Ag-high and -low modules, respectively, while collecting LECs only showed subtle differences in the expression of each. These results suggest an overall reduction in antigen archiving ability at 24 hours post infection, and this effect appears most prominent within the cLEC population.

To experimentally test the accuracy of these predictions, we infected mice in the footpad with WT CHIKV or CHIKV 181/25, an attenuated vaccine strain, which does not impair antigen acquisition by LN LECs^16^. After 24 hours, mice were immunized with 10 μg ova-488 in both calf muscles (20 μg/mouse total), and ova^+^ LECs were evaluated in the dLN 2 days later. As a positive control, mock-infected mice were immunized with 10 μg ova-488 and 5 μg polyI:C in both calf muscles. Consistent with our previous work, the percentage and number of ova^+^ LECs was similar between CHIKV 181/25-infected and polyI:C-treated mice (Fig. 5G–H). However, there was a significant reduction in the percentage and number of ova^+^ LECs in WT CHIKV-infected mice compared with CHIKV 181/25-infected mice (Fig. 5G–H). These data indicate that antigen acquisition, the first step in antigen archiving, is impaired during WT CHIKV infection as early as 24 hours after inoculation, prior to the alterations in LN LEC composition and LN cellular organization previously shown to occur during WT CHIKV infection^16,20^. Thus, these findings support the accuracy of our predictive models and further demonstrate how gene expression patterns can be used to inform LEC antigen archiving capability.

### A portable gene expression program for predicting antigen archiving

Using predictive models we were able to evaluate antigen archiving ability in mice that were not immunized with our antigen-DNA conjugates (Fig. 5, S8). We next sought to further assess the portability of these models (Fig. 2, S3, Table S2) by applying them to human datasets. To do this, we utilized previously published human scRNA-seq data for follicular lymphoma samples (FL) and metastasis-free control LN samples (MFLN)^43^. We first annotated LEC subsets using human reference data^44^ and were able to identify LEC subsets described previously^44^ (Figure S9A). To assess antigen archiving, we identified human homologs for our antigen archiving gene programs (Fig. 2, S3, Table S2). We then used the cLEC, fLEC, and collecting LEC models to predict antigen-archiving competent (Ag-competent) cells for each respective population. We find that the fraction of Ag-competent cLECs and fLECs (~ 0.5–0.75) is similar to what we observe in mice (Fig. 5C, S8C–D, S9B–C). However, comparison of MFLN and FL samples shows similar levels of predicted Ag-competent cells, suggesting that follicular lymphoma does not cause a significant reduction in the archiving ability of cLECs and fLECs (Figure S9B–C). Analysis of collecting LECs from these datasets shows very few predicated Ag-competent cells for both the MFLN and FL samples (Figure S9D). This indicates that either expression of the collecting LEC archiving program differs significantly between human and mouse or that human collecting LECs do not archive antigens in the same way or to the same level as mouse collecting LECs. Regardless, these findings demonstrate differences and similarities between human and mouse LN LECs. However, the functional consequences of this altered expression program on antigen archiving in human collecting LECs is still unclear and requires additional investigation. Overall these results illustrate the utility of our approach to investigate antigen archiving in human samples and other systems where inclusion of antigen-DNA conjugates is not feasible.

## Discussion

In this study, we defined a gene signature associated with LEC antigen archiving using an antigen conjugated to a DNA tag for use with single cell RNA sequencing. LECs acquire and archive foreign antigens beyond the peak of the primary immune response^8–11^. We previously demonstrated that antigen can be detected using a psDNA tag conjugated to a protein antigen for up to 14 days post-immunization, using single cell sequencing^11^. We further extended this timeline to 21 and 42 days post-immunization to assess the temporal dynamics of antigen archiving. Corroborating our published data, we found that antigens persist predominantly in LEC subsets, such as cLEC, fLEC, and collecting LEC, at late time points following both single and dual vaccinations (Fig. 1F, 3G). Notably, Ptx3 LECs capture antigens at day 2, but by day 14 antigen levels decline in these cells. One potential explanation for the loss of antigens within the Ptx3 LEC subset may be the lack of expression of *Cd274* (PDL1). PDL1 is an important regulator of LEC division and survival where loss of PD-L1 leads to increased LEC division^45^. Indeed, the LECs that undergo division during LN expansion following immunization are also the LECs that undergo apoptosis^45^. Thus, Ptx3 LECs, which have low expression of *CD274*^14^, likely undergo apoptosis and lose antigens earlier than the other LEC subsets during LN contraction.

Furthermore, we developed machine learning models to better predict cells capable of antigen archiving. The training of these classification models was based on the day 14 time point because it was an intermediate time point with robust antigen high and low cell populations. As a proof of concept, these models were able to predict the decrease in Ag-competent cells following CHIKV infection. Extensive data^16^ demonstrate how CHIKV infection disrupts the organization of the LN through interactions with LNSCs. We further tested the accuracy of these predictions by assessing the ability of LECs to archive fluorescent antigens in CHIKV-infected mice (Fig. 5). Thus, these classification models are an important advancement, as they enable the analysis of antigen archiving competency in esisting and future scRNA-seq datasets from different infections, immunizations, or disease states. This could be particularly important to identify which infections or inflammatory stimuli promote or hinder antigen archiving and could inform studies regarding when or which area to vaccinate. Upon expanding on our published study using antigen-DNA conjugates as a molecular tracking system, we demonstrated that the incorporation of two different antigen tags can accurately model the kinetics of antigen signals in different cell types using scRNA-seq. Furthermore, the generation of machine learning models to assess antigen archiving presents a new approach for future applications.

Genes associated with endosomal-lysosomal function were captured as part of this antigen archiving gene signatures. Lysosomal-associated genes in the Ag-high module (Figure S3A) could result from the large amount of antigen within the LECs. It seems likely that LECs trap foreign antigens as they pass through the LN similar to how LECs capture viral particles to prevent viral dissemination^17^. Ultimately, LECs must degrade or release antigens to neighboring cells, such as DCs. Antigen exchange between LECs and DCs is likely a by-product of the process of antigen archiving and has been maintained as small amounts of DC antigen presentation over a limited time period is likely an evolutionary benefit. Indeed, we have previously demonstrated an increased capacity for DC presentation of archived antigens during an unrelated infection^8^. During infection, the increased activation state of the DCs that acquire both antigens from the infection and antigens from LECs leads to improved memory T cell responses and increased effector function^8^. Alternatively, LECs could acquire antigens via different endocytic pathways. As an example, we and others have demonstrated that LEC endocytosis of proteins occurs via caveolin and clathrin mediated endocytosis *in vivo*^11^ and *in vitro*^46^. Caveosomes maintain a neutral pH and cargo can remain in caveosomes until transcytosis/recycling or lysosomal degradation via RAB5 dependent fusion with the early endosome^47^. It is possible that caveolin mediated endocytosis is one mechanism of antigen storage while clathrin mediated endocytosis targets excess antigen for degradation. The data presented here suggest that specific LECs are “pre-coded” to be competent in antigen archiving, particularly when our classification models were applied to naïve mice (Fig. 5). These models predict that certain LECs within naïve mice are predisposed to express the antigen-archiving gene signature (Figure S3C–D).

As LECs do not directly present archived antigens to CD8 T cells, there is a need for antigen exchange from LEC to migratory DC for the presentation of archived antigen-derived peptides^8,9,11^. Indeed, antigen-bearing LECs interact, particularly those that express ICAM1, and exchange antigen with DCs^9^. Building upon the live imaging of LEC-DC interactions to exchange archived antigens^9^, here we visualized the spatial localization of antigen bearing cells in the LN using the 10X Genomics Xenium and Nanostring GeoMx platforms. We confirmed by scRNA-seq that antigen signals within LEC and DC subsets correlated with our spatial data. We found that CCR7hi DCs and cDC2 T-bet DCs had antigen levels peaking at 14 days post-vaccination and that antigen levels dramatically decreased by day 21 post-vaccination (Fig. 1J). Interestingly, when we further extended this time course to 42 days, we still detected antigens in cDC2 Tbet- and CCR7hi DC populations (Fig. 1J). While it is unlikely that DCs are still acquiring and processing antigens from the initial immunization several weeks earlier, this finding supports the hypothesis that LECs are slowly releasing antigens over time through either direct LEC-DC interactions or via LEC apoptosis.

In dual-vaccinated mice, cLECs, fLECs, and collecting LECs have enhanced antigen acquisition and retention compared to mice receiving a single vaccination (Fig. 3D, G, S4B). The data we provide suggest: *i.* that LECs are capable of acquiring multiple antigens and *ii*. that LECs that already acquired one antigen are more effective at acquiring a second antigen. We found that the gene signature for LECs following one immunization is similar to the gene signature of LECs with multiple archived antigens. This suggests there are LECs that are susceptible to being reprogrammed for antigen acquisition and archiving (Ag-competent). It is possible that LNSCs, particularly LECs, undergo epigenetic reprogramming during an initial antigen encounter that provides increased responsiveness in regard to antigen acquisition. Trained immunity is thought to be mediated by epigenetic changes, alterations in gene expression, and metabolic reprogramming within innate immune cells. These changes lead to a heightened state of readiness and elimination of pathogens upon subsequent exposure^48,49^. Interestingly, this training is not limited to innate immune cells as one study showed that IFNb stimulation contributes to increased and quicker induction of interferon-stimulated genes (ISG) in mouse embryonic fibroblasts^50^. Upon restimulation, the rapid induction of ISGs was attributed to increased recruitment of RNA polymerase II to ISG loci and this was also associated with H3.3 and H3K36ME3 marks on the chromatin of ISG genes^50^. Thus, it is possible that LECs may undergo epigenetic modifications upon antigen acquisition to promote subsequent antigen uptake and retention.

We found higher antigen levels in both LEC subsets and DC subsets, CCR7hi DC and cDC2 Tbet-, upon a second antigen exposure when compared to single-immunized mice (Fig. 3D,G, S4A–C). This supports our published data demonstrating that migratory DCs^9^ and cDC1s are required for cross-presentation of archived antigens during the time frame of LEC apoptosis^8^. cDC1 cross-presentation of cell-associated antigens is partly attributed to their ability to internalize apoptotic cells or cell debris^51–54^. Therefore, when mice receive a second vaccination, some LECs undergo apoptosis, leading to the release of archived antigens that DCs can then acquire. Alternatively, DCs may have higher levels of antigen after a second exposure for the same reason as LECs. Myeloid progenitor cell populations do undergo innate training and thus perhaps DCs are also capable of increased antigen acquisition in addition to increased cytokine release as has been described^48,55,56^.

A caveat to our spatial transcriptomics studies is our inability to directly compare antigen signals from single- and dual- immunized mice. However, the Xenium and GeoMx spatial transcriptomics platforms lack the depth of scRNA-seq, making antigen detection sparse and limited by the ability of the probe to bind the target. To address this limitation we coupled these methods with scRNAseq datasets. Here, we found that LECs and DCs in the subcapsular sinus of the LN had the highest levels of antigen. These data support our finding that sequential vaccinations enhanced LEC antigen archiving and antigen exchange. Overall, this manuscript provides confirmatory evidence of our prior publications using transcriptional profiling and antigen-DNA tags. To build upon these data we provide a unique resource to assay antigen archiving capacity by identifying an antigen archiving gene signature and generating an antigen competency model to predict outcomes from other datasets, including those derived from human samples. Indeed, our analysis suggests a similar gene expression program is expressed in human LEC populations from existing datasets^44^, highlighting how processes like antigen archiving, defined in animal models of immunization, can be predictive of human biology.

## Materials and Methods

### Mice

5–6 week-old C57BL/6 mice were purchased from Charles River or Jackson Laboratory and bred and housed in the University of Colorado Anschutz Medical Campus Animal Facility. All animal procedures were approved by the Institutional Animal Care and Use Committee at the University of Colorado under protocol number 67.

### Immunizations and Infections

6–8-week-old C57BL/6 (CD45.2) mice were immunized with 1E4 plaque-forming units (PFU) of Vaccinia Western Reserve or 5 μg of poly I:C (Invivogen) with or without 5 μg of anti-CD40 (FGK4.5, BioXcell) and 20 μg of ova-psDNA or ova in 50 μL volume per footpad injection. Endotoxin levels were quantified using the Pierce Limulus Amebocyte Lysate Chromogenic Endotoxin Quantitation kit (Thermo Scientific) to be less than 0.5 EU/mg for either ova or ova conjugated to psDNA. For CHIKV infections, 4 week old C57BL/6 mice were inoculated with 10^3^ PFU of CHIKV 181/25 or CHIKV AF15561 (WT CHIKV) in a 10 μL volume in both rear footpads.

### Evaluation of antigen acquisition by flow cytometry

Antigen acquisition was evaluated using fluorescently labeled ovalbumin (ova) as previously described^8–11,16^ Ovalbumin (A5503, Sigma-Aldrich) was decontaminated of lipopolysaccharide using a Triton X-114 detoxification method and tested with Pierce LAL chromogenic endotoxin quantitation kit (88282, Thermo Fisher Scientific). Ovalbumin was labeled using an Alexafluor 488 succinimidyl ester labeling system (A20100, Thermo Fisher Scientific). CHIKV-infected mice were inoculated with 20 μg AF488-labeled ovalbumin via intramuscular injection into both calf muscles (10 μg per calf) 24 h after virus inoculation, and popliteal LNs were collected 2 days later for analysis of ova^+^ LNSCs by flow cytometry. As positive controls, mock-infected mice were inoculated with 20 μg AF488-labeled ovalbumin and 10 μg polyI:C via intramuscular injection into both calf muscles (10 μg ova-488 and 5 μg polyI:C per calf).

For preparation of single-cell suspensions for flow cytometry, the left and right popliteal LNs were combined for each mouse (2 LNs/sample), minced in Click’s media (Sigma-Aldrich) with 22G needles (Exelint), and digested for 1 hour at 37°C in 94 μg/mL DNase I (Roche) and 250 μg/mL collagenase type I and 250 μg/mL collagenase type IV (Worthington Biochemicals). Cell suspensions were passed through a 100 μm cell strainer (BD Falcon), and total viable cell numbers were enumerated by trypan blue exclusion. All single-cell suspensions were incubated for 15 minutes at 25°C in LIVE/DEAD Fixable Visolet Dead Cell Stain (Invitrogen) to identify viable cells and then stained for 45 minutes at 4°C with anti–mouse FcγRIII/ II (2.4G2; BD Pharmingen) and the following antibodies from BioLegend diluted in FACS buffer (PBS with 2% FBS): anti-CD45 (30-F11), anti-CD31 (clone 390), and anti-PDPN (8.1.1). Cells were washed 3 times in PBS/2% FBS and then fixed for 15 minutes in 1× PBS/1% PFA and analyzed on a BD LSR Fortessa cytometer using FACSDiva software. Analysis was performed using FlowJo software (Tree Star Inc.) and GraphPad Prism version 10.1.1. Data were evaluated for statistically significant differences using a 1-way ANOVA test followed by Tukey’s multiple-comparison test. *P* < 0.05 was considered statistically significant.

### Phosphorothioate oligonucleotides and conjugation to protein.

Oligonucleotides were synthesized by Integrated DNA Technologies (IDT) with phosphorothioated oligonucleotides at every linkage as previously described^11^. Oligonucleotides were conjugated to ovalbumin as previously described^11^ using iEDDA-click chemistry^57^. GeoMX tags: DNA tags were provided by IDT using standard desalting and 5’ Amino Modifier C6 with 70 phosphorothioate bonds. GeoMX Barcode 1 target sequence: 5’/5AmMC6/ GTTAGGAGGGTCCTTCTAATGTTAACGCCCGAATATTAGTCATATTTTGCTAGCGCCTATCAGCGTAAGA-3’; GeoMX Barcode 2 target sequence: 5’/5AmMC6/ GGCGATCCAGCCGGTATACCTTAGTCACATATACTATCGTAATATTGGCGGTTGCTGACAAGTAAATACG-3’. psDNA tags for single cell sequencing: psDNA2 5’/5AmMC6/ GACGTGTGCTCTTCCGATCTNNNCCTGAATTCGAGNNNGCTCACCTATTAGCGGCTAAGG/3Bio/ and psDNA4 5’/5AmMC6/ AGACGTGTGCTCTTCCGATCTNNNTCAGGTACCTGANNNGCTCACCTATTAGCGGCTAAGG/3Bio/ Endotoxin levels were quantified using the Pierce Limulus Amebocyte Lysate Chromogenic Endotoxin Quantitiation kit (ThermoScientific) to be less than 0.5EU/mg for either ovalbumin or ovalbumin conjugated to psDNA. Protein concentration was measured using the Pierce BCA Protein Assay kit (ThermoScientific) and confirmed on a protein SDS gel with silver stain.

### Preparation of single-cell suspensions

21 or 42 days following vaccination with 1E4 PFU of VV-WR and 20 μg of ova-psDNA per footpad, popliteal lymph nodes were harvested from 13 mice, with 4 lymph nodes mechanically separated with 22-gauge needles in 2 mL of EHAA media. Mouse popliteal lymph nodes were split into two digestion media’s. Eight popliteal LNs were digested in collagenase D (1mg/mL) and DNase 1 (0.25mg/mL) for 30 min at 37°C as described previously^9^ for isolation of the dendritic cells. The other 18 popliteal LNs were digested with 0.25 mg of Liberase DL (Roche, Indianapolis, IN) per mL of EHAA media with DNAse 1(Worthington, Lakewood, NJ) at 37°C to isolate the stromal cell population for 1 hr at 37°C with pipetting every 15 min to physically agitate the digested tissues. The stromal cell digestion media was replaced with fresh digestion media after 30 min. Following digestion, cells were filtered through a screen and washed with 5 mM EDTA in EHAA + 2.5% FBS (R&D Systems: S12450). The 8 popliteal LNs digested for dendritic cells isolation were then stained with CD11c (N418), CD11b (clone #M1/70) and B220 (clone #RA3–6B2), and a live/dead dye (564406, BD Biosciences). Live cells were then sorted into four tubes on a FACS Aria Cell Sorter (BD): sorted CD11c-APC Cy7 (clone N418 1:400) + cells, sorted CD11b PE-Cy7+ (clone M1/70,biolegend) cells, sorted B220 PE+ (clone RA3–6B2) cells and Fixable Viability Stain 510 (564406, BD Biosciences) ungated live cells, which were recombined at a 4:4:1:1 ratio, respectively. For the remaining lymph nodes in the stromal cell digest, cells were stained with CD45-PE (clone # 30-F11) and Ter119-PE (clone # TER-119) followed by magnetic bead isolation using the Miltenyi bead isolation kit. CD45-negative cells that passed through the column were then washed. Both sorted and selected (CD45 + and CD45-) cells were then washed with PBS in 0.1% BSA as described in the Cell Prep Guide (10x Genomics) and counted using a hemocytometer. Final concentration of cells was approximately 1600 cells/μL and approximately 10–20 μL were assayed.

### Single-cell library preparation using the 10x Genomics platform

Cells were assayed using the 10x Genomics single-cell 3’ expression kit v3 or v3 HT kit according to the manufacturer’s instructions (CG000053_CellPrepGuide_RevD) and CITE-seq protocol (cite-seq.com/protocol Cite-seq_109213) with the changes outlined in^11^ which include cDNA amplification and cleanup and amplification of antigen tag sequencing libraries. All libraries were sequenced on an Illumina NovaSeq 6000 with 2 × 150 base pair read lengths.

### Preparation of Spatial Transcriptomics Slides

The popliteal lymph nodes were harvested either 3 or 6 weeks after immunization with 20μg of one or both ova-psDNA GeoMx tags and 1e4 pfu per foot of vaccinia virus. Lymph nodes were fixed for 24-hours in 10% phosphate buffered formalin, and then placed in 70% Ethanol until embedding. The lymph nodes were embedded in paraffin wax, and (7 uM) sections were cut.

For analysis with the Nanostring GeoMx platform, 7μM sections were cut from paraffin-embedded LNs as described above. Slides were stained with Lyve-1–647 (clone: EPR21771), CD11c-Biotin (clone: D1V9Y), CD4 (clone: 4SM95). The nuclei were counterstained with SYTO^™^ 82 Orange Fluorescent Nucleic Acid Stain (S11363). Biotinylated antibodies were visualized with Streptavadin-AF594 (S32356) and the CD4 antibody was visualized with OPAL 520 (FP1487001KT, Akoya Biosciences). The ROI’s selected to undergo GeoMx DSP spatial transcriptomics were chosen based on Lyve-1 staining, CD11c staining and CD4 staining. Regions were selected based on the size of the area or the maximum size allowed for the R01.

For analysis with the 10x Genomics Xenium platform, 5 uM sections were cut from paraffin-embedded lymph nodes described above using a microtome. 2 LNs were analyzed for each immunization group (21 day tag, 42 day tag, 21 and 42 day tag). Sections were placed on 10x Genomics Xenium slides, baked for 2 hours at 60C, followed by deparaffinization and decrosslinking. Oligonucleotide probe panels were used that included the 379 gene Xenium Mouse Tissue Atlassing Panel (1000627, 10x Genomics) and a custom panel (1000561, 10x Genomics) that included our antigen DNA barcode sequences and additional genes identified from our scRNA-seq data. Probes were hybridized to mRNA and spatial barcode targets in-situ and rolling circle amplification (RCA) was used to boost fluorescent signal according to the manufacturer’s instructions. Tissue was imaged using the Xenium Analyzer (PN-1000569). Regions were manually defined, one region per lymph node, before capture. Each lymph node was captured in technical duplicate (two 5 uM sections per lymph node on separate Xenium slides). DAPI nuclear staining was used to identify individual cells in the automated Xenium Analyzer cell segmentation pipeline. Additional cell segmentation reagents (e.g. membrane dyes) were not applied.

### scRNA-seq gene expression processing

FASTQ files for the day 2 and day 14 time points were processed as previously described^11^. FASTQ files for the day 21, day 42, and dual immunized samples were processed using the Cell Ranger count pipeline (v6.0.1). Initial filtering of gene expression data was performed using the Seurat R package (v4.4.0). The CD45 + and CD45- samples were processed separately and were combined into separate Seurat objects that included all immunization conditions (2d, 14d, 21d, 42d, dual immunized). Cells were filtered based on the number of genes expressed (> 250 and < 5000) and the percentage of mitochondrial reads (< 20%). Genes were filtered to only include those detected in > 5 cells. Gene expression reads were normalized by the total mouse reads for the cell, multiplied by a scale factor of 10,000, and log-transformed (NormalizeData). Normalized mouse counts were scaled and centered (ScaleData) using the top 2,000 variable features (FindVariableFeatures). The scaled data were used for PCA (RunPCA) and the samples were integrated using the Harmony package^58^. The integrated data were then used to identify clusters (FindNeighbors, FindClusters) and run UMAP (RunUMAP).

For the CD45- samples, we used the integrated cell clusters to generate an initial set of broad cell type annotations using the R package clustifyr (v1.12.0)^21^ and reference data from Immgen^23^. These annotations were checked for accuracy and further refined using known cell type markers including Ptprc, Pdpn, Pecam1, Cd3e (T cells), Cd19 and Cd79a, (B cells), and Cdh1 and Krt8 (epithelial cells). To annotate LEC subsets, endothelial cells were filtered, re-integrated using the Harmony package^58^, and re-clustered. LEC subsets were classified using the clustifyr package along with previously published data^14^.

For the CD45 + samples, we used the integrated cell clusters to generate an initial set of cell type annotations using the R package clustifyr (v1.12.0) and combined reference data from Immgen^23^ along with published data for DC subsets^59^. These annotations were further refined using known cell type markers including Ptprc, Nkg7 (NK cells), Cd3e (T cells), and Cd19 (B cells). For visualization and downstream analysis, DCs were then filtered and re-integrated using the Harmony package^59^.

### Quantification of antigen signals

To compare antigen signals between samples, we calculated antigen scores by first diving antigen counts present in each cell by the total number of antigen UMI counts for the library (counts per million). To account for different levels of background signal present in each library, we calculated the 75th percentile of normalized counts for B and T cells for each library. We then used this value as an estimation of background signal since B and T cells should not take up antigen. The background signal estimated for each sample was then subtracted from the normalized counts for each cell. Background corrected values that were < 0 were set to 0. For visualization and downstream analysis, these background-corrected values were then log1p-transformed.

To identify antigen-high (Ag-high) and -low (Ag-low) cells, we used the calculated antigen scores for cLECs, fLECs, and collecting LECs from the day 14 time point. We used this time point since it is an intermediate time point in our time course and had distinct populations of Ag-high and Ag-low cells. We focused on cLECs, fLECs, and collecting LECs since they are the subsets with the highest overall antigen levels. For each subset we used k-means clustering to divide cells into two groups based on the day 14 antigen scores. To ensure consistent classification of Ag-high cells for each time point, we used the antigen score thresholds from the day 14 cells to identify Ag-high cells in the day 21 and day 42 time points. For the dual immunized samples, we identified Ag-high cells separately for the 21 day and 42 day antigen scores.

### Prediction of antigen archiving ability and identification of archiving gene modules

To identify gene expression modules associated with antigen archiving, we trained separate random forest classification models using the Ag-high and -low cells identified for day 14 cLECs, fLECs, and collecting LECs. To identify an initial set of features to use for training each model, we identified genes that were differentially expressed in Ag-high cells for at least one LEC subset from at least one of the time points. Differentially expressed genes were identified using the presto R package (https://github.com/immunogenomics/presto) and were filtered for those with an adjusted p value < Classification models were trained using the Ranger R package (v0.15.1)^60^. Cells from each LEC subset were split into equal training and testing groups. To identify optimal parameters for model training, a grid search was performed by varying the number of trees (num.trees), the number of variables to possibly split at each node (mtry), and the minimum node size to split at (min.node.size). In addition, the ratio of Ag-high and Ag-low cells used for training was also varied by randomly sampling these groups at different ratios. To identify features most predictive of antigen class for each model, features were filtered based on importance using a p-value cutoff of 0.05 and models were retrained. Ag-high and Ag-low gene modules were identified for each trained model by dividing features into two groups based on whether they were up- or downregulated in Ag-high cells. To identify optimal training parameters, model performance was assessed based on F1 score and balanced accuracy.

The optimized models for each LEC subset were used to predict Ag-high and -low cells in the 21 day, 42 day, and dual immunized samples. Based on the predicted antigen classes, cells were divided into three groups, 1) has high levels of antigen based on quantified antigen scores (Ag-high), 2) has low levels of antigen but is predicted to be Ag-high (Ag-competent), or 3) has low levels of antigen (Ag-low). For the dual immunized mice, cells were divided into four groups, 1) classified as Ag-high for both antigens (double-high), 2) classified as Ag-high for only one antigen (single-high), 3) classified as Ag-low for both antigens but predicted to be Ag-high (Ag-competent), or 4) classified as Ag-low for both antigens (Ag-low). To compare expression of the Ag-high and Ag-low gene modules derived from each model, module scores were calculated using the Seurat package (AddModuleScore). To identify cell processes that could influence antigen archiving, gene ontology terms were identified for the Ag-high and Ag-low gene modules for each LEC subset using the clusterProfiler R package. Ontology terms were filtered to only include those with > 10 genes, < 1000 genes, and an adjusted p value < 0.05.

Antigen archiving ability was assessed for mock- and CHIKV-infected samples (3 biological replicates each) using previously published data^16,17^. These data were processed and cell types were annotated as previously described^16^. Ag-competent cells were predicted using the classification models trained using day 14 cLECs, fLECs, and collecting LECs as described above. Antigen archiving ability was assessed for mock- vs CHIKV-infected mice by comparing the fraction of predicted Ag-competent cells for each biological replicate.

### Assessing antigen archiving in follicular lymphoma

scRNA-seq count matrices for human follicular lymphoma samples and metastasis-free lymph node samples^43^ were downloaded and processed using the Seurat R package (NormalizeData, FindVariableFeatures, ScaleData, RunPCA, RunUMAP). Samples were integrated using the Harmony R package^58^. LEC subsets were annotated using the clustifyr R package^21^ and published reference data for human LEC populations^44^. To identify predicted antigen archiving competent cells, we then identified human homologs for our Ag-low and Ag-high gene programs. We then predicted Ag-competent cells for human cLECs, fLECs, and collecting LECs using gene expression data for the homologous genes and the corresponding random forest models described previously (Fig. 2). Since the prediction models require data for all genes used for training, genes with no identifiable human homolog were included as zeros (13%). We then calculated the fraction of cells predicted to be Ag-competent for each LEC subset for samples with > 20 cells.

### Processing of Xenium data

Data for each LN tissue section was processed as a separate “field of view” (FOV) using the Seurat R package (v4.4.0). For each slide a separate Seurat object was generated for each FOV. Antigen tag counts were added to the objects as a separate assay. Gene expression counts for each cell were normalized (SCTransform), and PCA and UMAP were performed (RunPCA, RunUMAP) followed by clustering (FindNeighbors, FindClusters). To annotate cell types, we first generated a combined cell type reference using our scRNA-seq data for CD45 + and CD45- cells from the day 42 immunized mice (Fig. 1). To do this we combined the CD45 + and CD45- datasets and reprocessed using SCTransform. We then transferred cell type labels to each Xenium FOV object using Seurat (FindTransferAnchors, TransferData). These initial annotations were further refined using common marker genes including Ptprc, Cd19, and Cd3d. Antigen + cells were identified for each cell type as any cell with > 0 counts for either the 21 day or 42 day antigen barcode. To quantify the distance between DCs and Ag + LECs, sf objects were generated using the cell boundary coordinates for each cell and the distance between each DC and each Ag + LEC was calculated using the sf R package.

### Processing of GeoMx DSP data

Sequencing libraries generated from the Nanostring GeoMx DSP platform were sequenced on an Illumina NovaSeq6000 at a depth of 200 million reads. FASTQ files were processed using the GeoMx NGS Pipeline v2.0 software. Downstream processing and analysis was performed following best practices described by Nanostring using the GeomxTools (v3.4.0) R package. Data were filtered to only include segments with > 1000 aligned reads, > 75% of reads aligned, > 80% of reads stitched, > 80% of reads trimmed, and > 100 estimated nuclei. Probes were filtered to remove outlier probes based on the Grubb’s test and aggregated for each gene. Genes were filtered to remove those detected in < 1% of segments. For gene expression data, counts were normalized based on the 75th percentile of counts present in each segment. Antigen counts were normalized based on background signals estimated using counts for negative control probes. To allow the 21 day and 42 day antigen signals to be compared together on the same plot, signals were scaled separately for each antigen (relative Ag signal, Figure S7C). To do this z-scores were calculated for the 21 day antigen signal for all regions from the 21 day and dual immunized mice, and 42 day antigen signal for all regions from the 42 day and dual immunized mice.

## Supplementary Material

Supplement 1

## Data Availability

All data and material within this article will be available upon reasonable request to the corresponding author.

## References

[1] Kim T.S., Hufford M.M., Sun J., Fu Y.X., and Braciale T.J. (2010). Antigen persistence and the control of local T cell memory by migrant respiratory dendritic cells after acute virus infection. J Exp Med 207, 1161–1172. jem.20092017 [pii] 10.1084/jem.20092017. 20513748 PMC2882836

[2] Kim T.S., Sun J., and Braciale T.J. (2011). T cell responses during influenza infection: getting and keeping control. Trends Immunol 32, 225–231. S1471–4906(11)00041-X [pii] 10.1016/j.it.2011.02.006. 21435950 PMC3090469

[3] Takamura S., Roberts A.D., Jelley-Gibbs D.M., Wittmer S.T., Kohlmeier J.E., and Woodland D.L. (2010). The route of priming influences the ability of respiratory virus-specific memory CD8+ T cells to be activated by residual antigen. J Exp Med 207, 1153–1160. jem.20090283 [pii] 10.1084/jem.20090283. 20457758 PMC2882830

[4] Zammit D.J., Cauley L.S., Pham Q.M., and Lefrancois L. (2005). Dendritic cells maximize the memory CD8 T cell response to infection. Immunity 22, 561–570. S1074–7613(05)00096–8 [pii] 10.1016/j.immuni.2005.03.005. 15894274 PMC2857562

[5] Zammit D.J., Turner D.L., Klonowski K.D., Lefrancois L., and Cauley L.S. (2006). Residual antigen presentation after influenza virus infection affects CD8 T cell activation and migration. Immunity 24, 439–449. 10.1016/j.immuni.2006.01.015. 16618602 PMC2861289

[6] Jelley-Gibbs D.M., Brown D.M., Dibble J.P., Haynes L., Eaton S.M., and Swain S.L. (2005). Unexpected prolonged presentation of influenza antigens promotes CD4 T cell memory generation. J Exp Med 202, 697–706. jem.20050227 [pii] 10.1084/jem.20050227. 16147980 PMC2212871

[7] Woodland D.L., and Kohlmeier J.E. (2009). Migration, maintenance and recall of memory T cells in peripheral tissues. Nat Rev Immunol 9, 153–161. nri2496 [pii] 10.1038/nri2496. 19240755

[8] Doan T.A., Forward T.S., Schafer J.B., Lucas E.D., Fleming I., Uecker-Martin A., Ayala E., Guthmiller J.J., Hesselberth J.R., Morrison T.E., and Tamburini B.A.J. (2024). Immunization-induced antigen archiving enhances local memory CD8+ T cell responses following an unrelated viral infection. NPJ Vaccines 9, 66. 10.1038/s41541-024-00856-6. 38514656 PMC10957963

[9] Kedl R.M., Lindsay R.S., Finlon J.M., Lucas E.D., Friedman R.S., and Tamburini B.A.J. (2017). Migratory dendritic cells acquire and present lymphatic endothelial cell-archived antigens during lymph node contraction. Nat Commun 8, 2034. 10.1038/s41467-017-02247-z. 29229919 PMC5725486

[10] Tamburini B.A., Burchill M.A., and Kedl R.M. (2014). Antigen capture and archiving by lymphatic endothelial cells following vaccination or viral infection. Nat Commun 5, 3989. 10.1038/ncomms4989. 24905362 PMC4073648

[11] Walsh S.M., Sheridan R.M., Lucas E.D., Doan T.A., Ware B.C., Schafer J., Fu R., Burchill M.A., Hesselberth J.R., and Tamburini B.A.J. (2021). Molecular tracking devices quantify antigen distribution and archiving in the murine lymph node. Elife 10. 10.7554/eLife.62781. PMC8116055 33843587

[12] Rodda L.B., Lu E., Bennett M.L., Sokol C.L., Wang X., Luther S.A., Barres B.A., Luster A.D., Ye C.J., and Cyster J.G. (2018). Single-Cell RNA Sequencing of Lymph Node Stromal Cells Reveals Niche-Associated Heterogeneity. Immunity 48, 1014–1028 e1016. 10.1016/j.immuni.2018.04.006. 29752062 PMC5971117

[13] Takeda A., Salmi M., and Jalkanen S. (2023). Lymph node lymphatic endothelial cells as multifaceted gatekeepers in the immune system. Trends Immunol 44, 72–86. 10.1016/j.it.2022.10.010. 36463086

[14] Xiang M., Grosso R.A., Takeda A., Pan J., Bekkhus T., Brulois K., Dermadi D., Nordling S., Vanlandewijck M., Jalkanen S., (2020). A Single-Cell Transcriptional Roadmap of the Mouse and Human Lymph Node Lymphatic Vasculature. Front Cardiovasc Med 7, 52. 10.3389/fcvm.2020.00052. 32426372 PMC7204639

[15] Golas G., Alonso J.D., and Toth Z. (2019). Characterization of de novo lytic infection of dermal lymphatic microvascular endothelial cells by Kaposi’s sarcoma-associated herpesvirus. Virology 536, 27–31. 10.1016/j.virol.2019.07.028. 31394409 PMC6733618

[16] Lucas C.J., Sheridan R.M., Reynoso G.V., Davenport B.J., McCarthy M.K., Martin A., Hesselberth J.R., Hickman H.D., Tamburini B.A., and Morrison T.E. (2024). Chikungunya virus infection disrupts lymph node lymphatic endothelial cell composition and function via MARCO. JCI Insight 9. 10.1172/jci.insight.176537. PMC11143926 38194268

[17] Carpentier K.S., Sheridan R.M., Lucas C.J., Davenport B.J., Li F.S., Lucas E.D., McCarthy M.K., Reynoso G.V., May N.A., Tamburini B.A.J., (2021). MARCO(+) lymphatic endothelial cells sequester arthritogenic alphaviruses to limit viremia and viral dissemination. EMBO J 40, e108966. 10.15252/embj.2021108966. 34618370 PMC8591538

[18] Zhang R., Earnest J.T., Kim A.S., Winkler E.S., Desai P., Adams L.J., Hu G., Bullock C., Gold B., Cherry S., and Diamond M.S. (2019). Expression of the Mxra8 Receptor Promotes Alphavirus Infection and Pathogenesis in Mice and Drosophila. Cell Rep 28, 2647–2658 e2645. 10.1016/j.celrep.2019.07.105. 31484075 PMC6745702

[19] Zhang R., Kim A.S., Fox J.M., Nair S., Basore K., Klimstra W.B., Rimkunas R., Fong R.H., Lin H., Poddar S., (2018). Mxra8 is a receptor for multiple arthritogenic alphaviruses. Nature 557, 570–574. 10.1038/s41586-018-0121-3. 29769725 PMC5970976

[20] McCarthy M.K., Davenport B.J., Reynoso G.V., Lucas E.D., May N.A., Elmore S.A., Tamburini B.A., Hickman H.D., and Morrison T.E. (2018). Chikungunya virus impairs draining lymph node function by inhibiting HEV-mediated lymphocyte recruitment. JCI Insight 3. 10.1172/jci.insight.121100. PMC6124534 29997290

[21] Fu R., Gillen A.E., Sheridan R.M., Tian C., Daya M., Hao Y., Hesselberth J.R., and Riemondy K.A. (2020). clustifyr: an R package for automated single-cell RNA sequencing cluster classification. F1000Res 9, 223. 10.12688/f1000research.22969.2. 32765839 PMC7383722

[22] Shay T., and Kang J. (2013). Immunological Genome Project and systems immunology. Trends Immunol 34, 602–609. 10.1016/j.it.2013.03.004. 23631936 PMC4615706

[23] Malhotra D., Fletcher A.L., Astarita J., Lukacs-Kornek V., Tayalia P., Gonzalez S.F., Elpek K.G., Chang S.K., Knoblich K., Hemler M.E., (2012). Transcriptional profiling of stroma from inflamed and resting lymph nodes defines immunological hallmarks. Nat Immunol 13, 499–510. 10.1038/ni.2262. 22466668 PMC3366863

[24] Kamath A.T., Henri S., Battye F., Tough D.F., and Shortman K. (2002). Developmental kinetics and lifespan of dendritic cells in mouse lymphoid organs. Blood 100, 1734–1741. 12176895

[25] Mijanovic O., Petushkova A.I., Brankovic A., Turk B., Solovieva A.B., Nikitkina A.I., Bolevich S., Timashev P.S., Parodi A., and Zamyatnin A.A. Jr. (2021). Cathepsin D-Managing the Delicate Balance. Pharmaceutics 13. 10.3390/pharmaceutics13060837. PMC8229105 34198733

[26] Hasan S., Fernandopulle M.S., Humble S.W., Frankenfield A.M., Li H., Prestil R., Johnson K.R., Ryan B.J., Wade-Martins R., Ward M.E., and Hao L. (2023). Multi-modal proteomic characterization of lysosomal function and proteostasis in progranulin-deficient neurons. Mol Neurodegener 18, 87. 10.1186/s13024-023-00673-w. 37974165 PMC10655356

[27] Santos M.N., Paushter D.H., Zhang T., Wu X., Feng T., Lou J., Du H., Becker S.M., Fragoza R., Yu H., and Hu F. (2022). Progranulin-derived granulin E and lysosome membrane protein CD68 interact to reciprocally regulate their protein homeostasis. J Biol Chem 298, 102348. 10.1016/j.jbc.2022.102348. 35933009 PMC9450144

[28] Kumamoto K., Stewart T.A., Johnson A.R., and Erdos E.G. (1981). Prolylcarboxypeptidase (angiotensinase C) in human lung and cultured cells. J Clin Invest 67, 210–215. 10.1172/JCI110015. 7451650 PMC371589

[29] He Y., Kaya I., Shariatgorji R., Lundkvist J., Wahlberg L.U., Nilsson A., Mamula D., Kehr J., Zareba-Paslawska J., Biverstal H., (2023). Prosaposin maintains lipid homeostasis in dopamine neurons and counteracts experimental parkinsonism in rodents. Nat Commun 14, 5804. 10.1038/s41467-023-41539-5. 37726325 PMC10509278

[30] Pamarthy S., Kulshrestha A., Katara G.K., and Beaman K.D. (2018). The curious case of vacuolar ATPase: regulation of signaling pathways. Mol Cancer 17, 41. 10.1186/s12943-018-0811-3. 29448933 PMC5815226

[31] Song Q., Meng B., Xu H., and Mao Z. (2020). The emerging roles of vacuolar-type ATPase-dependent Lysosomal acidification in neurodegenerative diseases. Transl Neurodegener 9, 17. 10.1186/s40035-020-00196-0. 32393395 PMC7212675

[32] Popova N.V., Deyev I.E., and Petrenko A.G. (2013). Clathrin-mediated endocytosis and adaptor proteins. Acta Naturae 5, 62–73. PMC3848845 24307937

[33] Bonilha C.S., Benson R.A., Brewer J.M., and Garside P. (2020). Targeting Opposing Immunological Roles of the Junctional Adhesion Molecule-A in Autoimmunity and Cancer. Front Immunol 11, 602094. 10.3389/fimmu.2020.602094. 33324419 PMC7723963

[34] Takeda Y., Matoba K., Kawanami D., Nagai Y., Akamine T., Ishizawa S., Kanazawa Y., Yokota T., and Utsunomiya K. (2019). ROCK2 Regulates Monocyte Migration and Cell to Cell Adhesion in Vascular Endothelial Cells. Int J Mol Sci 20. 10.3390/ijms20061331. PMC6471293 30884801

[35] Singh S.K., Roy R., Kumar S., Srivastava P., Jha S., Rana B., and Rana A. (2023). Molecular Insights of MAP4K4 Signaling in Inflammatory and Malignant Diseases. Cancers (Basel) 15. 10.3390/cancers15082272. PMC10136566 37190200

[36] Lee N., Kim D., and Kim W.U. (2019). Role of NFAT5 in the Immune System and Pathogenesis of Autoimmune Diseases. Front Immunol 10, 270. 10.3389/fimmu.2019.00270. 30873159 PMC6401628

[37] Garcia-Alonso S., Romero-Perez I., Gandullo-Sanchez L., Chinchilla L., Ocana A., Montero J.C., and Pandiella A. (2021). Altered proTGFalpha/cleaved TGFalpha ratios offer new therapeutic strategies in renal carcinoma. J Exp Clin Cancer Res 40, 256. 10.1186/s13046-021-02051-0. PMC8365933 34399807

[38] Chang W., Gao W., Liu D., Luo B., Li H., Zhong L., and Chen Y. (2024). The upregulation of TGM2 is associated with poor prognosis and the shaping of the inflammatory tumor microenvironment in lung squamous cell carcinoma. Am J Cancer Res 14, 2823–2838. 10.62347/OBES4130. 39005693 PMC11236791

[39] Han L., Zhang M., Wang M., Jia J., Zhao M., Fan Y., and Li X. (2016). High Mobility Group Box-1 Promotes Inflammation-Induced Lymphangiogenesis via Toll-Like Receptor 4-Dependent Signalling Pathway. PLoS One 11, e0154187. 10.1371/journal.pone.0154187. 27100831 PMC4839690

[40] Lutter S., and Makinen T. (2014). Regulation of lymphatic vasculature by extracellular matrix. Adv Anat Embryol Cell Biol 214, 55–65. 10.1007/978-3-7091-1646-3_5. 24276886

[41] Lutter S., Xie S., Tatin F., and Makinen T. (2012). Smooth muscle-endothelial cell communication activates Reelin signaling and regulates lymphatic vessel formation. J Cell Biol 197, 837–849. 10.1083/jcb.201110132. 22665518 PMC3373399

[42] Mueller S.N., Hosiawa-Meagher K.A., Konieczny B.T., Sullivan B.M., Bachmann M.F., Locksley R.M., Ahmed R., and Matloubian M. (2007). Regulation of homeostatic chemokine expression and cell trafficking during immune responses. Science 317, 670–674. 10.1126/science.1144830. 17673664

[43] Abe Y., Sakata-Yanagimoto M., Fujisawa M., Miyoshi H., Suehara Y., Hattori K., Kusakabe M., Sakamoto T., Nishikii H., Nguyen T.B., (2022). A single-cell atlas of non-haematopoietic cells in human lymph nodes and lymphoma reveals a landscape of stromal remodelling. Nat Cell Biol 24, 565–578. 10.1038/s41556-022-00866-3. 35332263 PMC9033586

[44] Takeda A., Hollmen M., Dermadi D., Pan J., Brulois K.F., Kaukonen R., Lonnberg T., Bostrom P., Koskivuo I., Irjala H., (2019). Single-Cell Survey of Human Lymphatics Unveils Marked Endothelial Cell Heterogeneity and Mechanisms of Homing for Neutrophils. Immunity 51, 561–572 e565. 10.1016/j.immuni.2019.06.027. 31402260

[45] Lucas E.D., Finlon J.M., Burchill M.A., McCarthy M.K., Morrison T.E., Colpitts T.M., and Tamburini B.A.J. (2018). Type 1 IFN and PD-L1 Coordinate Lymphatic Endothelial Cell Expansion and Contraction during an Inflammatory Immune Response. J Immunol 201, 1735–1747. 10.4049/jimmunol.1800271. 30045970 PMC6125167

[46] Triacca V., Guc E., Kilarski W.W., Pisano M., and Swartz M.A. (2017). Transcellular Pathways in Lymphatic Endothelial Cells Regulate Changes in Solute Transport by Fluid Stress. Circ Res 120, 1440–1452. 10.1161/CIRCRESAHA.116.309828. 28130294

[47] Pelkmans L., Kartenbeck J., and Helenius A. (2001). Caveolar endocytosis of simian virus 40 reveals a new two-step vesicular-transport pathway to the ER. Nat Cell Biol 3, 473–483. 10.1038/35074539. 11331875

[48] Netea M.G., Dominguez-Andres J., Barreiro L.B., Chavakis T., Divangahi M., Fuchs E., Joosten L.A.B., van der Meer J.W.M., Mhlanga M.M., Mulder W.J.M., (2020). Defining trained immunity and its role in health and disease. Nat Rev Immunol 20, 375–388. 10.1038/s41577-020-0285-6. 32132681 PMC7186935

[49] Netea M.G., Joosten L.A., Latz E., Mills K.H., Natoli G., Stunnenberg H.G., O’Neill L.A., and Xavier R.J. (2016). Trained immunity: A program of innate immune memory in health and disease. Science 352, aaf1098. 10.1126/science.aaf1098. PMC5087274 27102489

[50] Kamada R., Yang W., Zhang Y., Patel M.C., Yang Y., Ouda R., Dey A., Wakabayashi Y., Sakaguchi K., Fujita T., (2018). Interferon stimulation creates chromatin marks and establishes transcriptional memory. Proc Natl Acad Sci U S A 115, E9162–E9171. 10.1073/pnas.1720930115. 30201712 PMC6166839

[51] Belz G.T., Shortman K., Bevan M.J., and Heath W.R. (2005). CD8alpha+ dendritic cells selectively present MHC class I-restricted noncytolytic viral and intracellular bacterial antigens in vivo. J Immunol 175, 196–200. 10.4049/jimmunol.175.1.196. 15972648 PMC2778481

[52] Cabeza-Cabrerizo M., Cardoso A., Minutti C.M., Pereira da Costa M., and Reis e Sousa C. (2021). Dendritic Cells Revisited. Annu Rev Immunol 39, 131–166. 10.1146/annurev-immunol-061020-053707. 33481643

[53] Desch A.N., Randolph G.J., Murphy K., Gautier E.L., Kedl R.M., Lahoud M.H., Caminschi I., Shortman K., Henson P.M., and Jakubzick C.V. (2011). CD103+ pulmonary dendritic cells preferentially acquire and present apoptotic cell-associated antigen. J Exp Med 208, 1789–1797. 10.1084/jem.20110538. 21859845 PMC3171085

[54] Schulz O., and Reis e Sousa C. (2002). Cross-presentation of cell-associated antigens by CD8alpha+ dendritic cells is attributable to their ability to internalize dead cells. Immunology 107, 183–189. 10.1046/j.1365-2567.2002.01513.x. 12383197 PMC1782783

[55] Quintin J., Saeed S., Martens J.H.A., Giamarellos-Bourboulis E.J., Ifrim D.C., Logie C., Jacobs L., Jansen T., Kullberg B.J., Wijmenga C., (2012). Candida albicans infection affords protection against reinfection via functional reprogramming of monocytes. Cell Host Microbe 12, 223–232. 10.1016/j.chom.2012.06.006. 22901542 PMC3864037

[56] Saeed S., Quintin J., Kerstens H.H., Rao N.A., Aghajanirefah A., Matarese F., Cheng S.C., Ratter J., Berentsen K., van der Ent M.A., (2014). Epigenetic programming of monocyte-to-macrophage differentiation and trained innate immunity. Science 345, 1251086. 10.1126/science.1251086. 25258085 PMC4242194

[57] van Buggenum J.A., Gerlach J.P., Eising S., Schoonen L., van Eijl R.A., Tanis S.E., Hogeweg M., Hubner N.C., van Hest J.C., Bonger K.M., and Mulder K.W. (2016). A covalent and cleavable antibody-DNA conjugation strategy for sensitive protein detection via immuno-PCR. Sci Rep 6, 22675. 10.1038/srep22675. 26947912 PMC4780193

[58] Korsunsky I., Millard N., Fan J., Slowikowski K., Zhang F., Wei K., Baglaenko Y., Brenner M., Loh P.R., and Raychaudhuri S. (2019). Fast, sensitive and accurate integration of single-cell data with Harmony. Nat Methods 16, 1289–1296. 10.1038/s41592-019-0619-0. 31740819 PMC6884693

[59] Brown C.C., Gudjonson H., Pritykin Y., Deep D., Lavallee V.P., Mendoza A., Fromme R., Mazutis L., Ariyan C., Leslie C., (2019). Transcriptional Basis of Mouse and Human Dendritic Cell Heterogeneity. Cell 179, 846–863 e824. 10.1016/j.cell.2019.09.035. 31668803 PMC6838684

[60] Wright M.N., and Ziegler A. (2017). ranger: A Fast Implementation of Random Forests for High Dimensional Data in C++ and R. Journal of Statistical Software 77, 1–17. 10.18637/jss.v077.i01.

