# Supplementary material for: A specific gene expression program underlies antigen archiving by lymphatic endothelial cells in mammalian lymph nodes
Source: Res Sq. 2024 Dec 10:rs.3.rs-5493746. Preprint. [Version 1] doi: 10.21203/rs.3.rs-5493746/v1 (PMC11661310; doi:10.21203/rs.3.rs-5493746/v1)
Supplement: Supplement 1 [file NIHPPRS5493746V1-supplement-1.pdf]

Supplementary Files

This is a list of supplementary files associated with this preprint. Click to download.

- Sheridanetalsupplementalfigures.pdf
